# Gut Bacteria of *Columbia livia* Are a Potential Source of Anti-Tumour Molecules

**DOI:** 10.31557/APJCP.2021.22.3.733

**Published:** 2021-03

**Authors:** Morhanavallee Soopramanien, Naveed Ahmed Khan, Bibi Noorheen Haleema Mooneerah Neerooa, Kuppusamy Sagathevan, Ruqaiyyah Siddiqui

**Affiliations:** 1 *Department of Biological Sciences, Sunway University, Bandar Sunway, Malaysia. *; 2 *Department of Clinical Sciences, College of Medicine, University of Sharjah, University City, Sharjah, United Arab Emirates. *; 3 *College of Arts and Sciences, American University of Sharjah, University City, Sharjah, United Arab Emirates. *

**Keywords:** Rock pigeon, Columba livia domestica, gastrointestinal microbiota, anticancer

## Abstract

**Objectives:**

The overall aim was to determine whether gut bacteria of Columbia livia are a potential source of antitumour molecules.

**Methods:**

Faecal and gut microbiota of Columbia livia were isolated, identified and conditioned media were prepared containing metabolites. Growth inhibition, lactate dehydrogenase cytotoxicity and cell survival assays were accomplished against cervical cancer cells. Next, liquid-chromatography mass spectrometry was conducted to elucidate the molecules present.

**Results:**

A plethora of bacteria from faecal matter and gastrointestinal tract were isolated. Selected conditioned media exhibited potent anticancer effects and displayed cytotoxicity to cervical cancer cells at IC_50_ concentration of 10.65 and 15.19 µg/ml. Moreover, cells treated with conditioned media exhibited morphological changes, including cell shrinking and rounding; indicative of apoptosis, when compared to untreated cells. A total of 111 and 71 molecules were revealed from these gut and faecal metabolites. The identity of 60 molecules were revealed including, dihydroxymelphalan. Nonetheless, 122 molecules remain unidentified and are the subject of future studies.

**Conclusion:**

These findings suggest that gut bacteria of Columbia livia possess molecules, which may have anticancer activities. Further in silico testing and/or high throughput screening will determine potential anticancer properties of these molecules.

## Introduction

Cancer remains a leading cause of mortality worldwide despite advances in the development of innovative therapeutic options, such as chemotherapy, radiation therapy, stem cell therapy, immunotherapy, targeted therapy, hormone therapy and surgery (Khan et al., 2019; Chakraborty and Rahman, 2012), suggesting the need to identify novel therapeutic agents. Notably, species such as crocodiles live in environments laden with heavy metals, feed on rotten meat, often tolerate high levels of radiation, are long-lived but rarely reported to develop cancer (Jeyamogan et al., 2017; 2019; Siddiqui et al., 2017; Siddiqui et al., 2016). Other species such as cockroaches exhibit high radiation resistance (lethal dose is 15 times higher than for humans) (Mosaheb et al., 2018; Soopramanien et al., 2019). Thus it is interesting to understand the mechanisms by which such hardy species are able to endure carcinogenic agents that are detrimental to humans. In support, our previous studies have shown that gut bacteria of crocodiles, water monitor lizard and scorpion possess potential anticancer molecules (Mosaheb et al., 2018; Soopramanien et al., 2019; Heyde and Ruder, 2015). For the first time, here we tested gut bacteria of pigeon (Columbia livia) for antitumour effects. 

## Materials and Methods

*Ethics committee approval*

Ethics approval was obtained from Sunway Research Ethics Committee (Research Ethics Approval Code: PGSUNREC 2019/023). The animal was procured from the wild, species identified, handled, and dissected by qualified zoologist, Dr K Sagathevan who routinely performs such procedures. 

*Gut and Faecal sample collection*

The gastrointestinal tract was carefully removed and opened with a longitudinal incision. The gut bacteria were isolated using sterile cotton swabs and were then inoculated on nutrient agar and blood agar plates. Bacteria from the faeces were isolated using sterile cotton swab and plated as mentioned above. Plates were incubated overnight at 37^o^C and bacterial colonies were subjected to identification as described previously (Soopramanien et al., 2019; Akbar et al., 2018). 

*Bacterial identification*

Bacterial identification was carried out based on their texture, size, colour, and shape and inoculating them onto separate fresh nutrient agar plates which were successively incubated overnight at 37oC. Bacteria were subcultured until agars with pure cultures were obtained. Pure bacteria were subjected to Gram staining. Next, bacterial identification was conducted using Analytical profile index (API) identification strip; API 20E was used for gram-negative bacteria while API staph was used for gram-positive bacteria as described previously (Soopramanien et al., 2019; Akbar et al., 2018).

*Conditioned medium (CM) preparation*

Pure bacterial cultures were inoculated in RPMI-1640 were incubated for 48h, at 37^o^C. Following incubation, bacterial cultures were centrifuged at 4^o^C, 10,000 x g, for 1h. Supernatant were collected and filtered using sterile 0.22μm pore size cellulose acetate syringe filter, also referred to CM (conditioned media). The CM were quantified for protein estimation using Bradford assay and then stored at -80^o^C (Soopramanien et al., 2019; Akbar et al., 2018). 

*Culture of cancer and normal cells*

The cancer cells used in this study were Cervical cancer (HeLa (ATCC^®^ CCL2™)), breast cancer (MCF7 (ATCC^®^ HTB-22™)) and prostate cancer (PC3 (ATCC^® ^CRL1435™)) and normal cell line; aneuploid immortal keratinocyte (HaCaT). Cells were cultured in RPMI-1640 augmented with 10% foetal bovine serum (FBS), 1% L-glutamine, 1% penicillin streptomycin antibiotic and 1% Minimum Essential Media (MEM) Non-Essential amino acid at 37oC with 5% carbon dioxide and 95% humidity (Soopramanien et al., 2019; Akbar et al., 2018).

*Growth inhibition assays*

Assays to determine inhibition of growth was performed as previously described. Briefly, cells were grown in 96-well plates until 50% confluency was reached. Control wells were trypsinised to determine the cell count, while experimental wells were treated with CM prepared from bacteria isolated from both faeces and gut of C. livia. CM from E. coli K-12 (a non-pathogenic laboratory strain) was used as negative control. The treated cells were incubated at 37oC in a 5% CO_2_ with 95% humidity, until the untreated cells became 100% confluent. The cells were then trypsinised with 2.5% trypsin for 15 min and subjected to Trypan blue exclusion assay using a haemocytometer. The growth inhibition effects were established by comparing the number of viable cells of untreated cells (control) and treated cells. To confirm the anticancer activity of the CM, selected CM were concentrated using vacuum concentrator. CM were then quantified through Bradford assay as described above and used for growth inhibitory assays at a concentration of 10µg/ml.

*Cytotoxicity assays, cell staining and survival assays*

Cells were grown to confluency in 96-wells plates. Next, cells were treated with CM prepared from bacteria isolated from faeces and gut of C. livia for 24 h at 37^o^C in a 5% CO_2_ with 95% humidity. CM from E. coli K-12 was used as negative control, while the positive control was prepared by treating control cells with 0.2% Triton X-100 for 30 min at 37°C, to induce 100% cell death. Next, the supernatants were collected and lactate dehydrogenase release was determined using cytotoxicity detection kit. The percentage cytotoxicity was calculated as follows:

% cytotoxicity = ((Absorbancesample – Absorbancenegative control)/ (Absorbancepositive control – Absorbancenegative control)) X 100, whereby the negative control comprised of cells treated with RPMI-1640 media only, and the positive control contained cells treated with the detergent: Triton X-100.

Moreover, cell staining was performed. Briefly, cells were fixed with 100% acetone and 100% methanol in a 1:1 ratio for 15min and stained with 0.4% Trypan blue for 15min. Plate was dried and pictures of individual wells were captured. Cell survival assay was also conducted to determine viability of cells treated with CM. Briefly, cells treated with CM were collected and seeded onto new plates containing growth media for 24 h at 37^o^C in a 5% CO_2_ with 95% humidity, and their re-growth were examined using a light microscope (Soopramanien et al., 2019; Akbar et al., 2018).

*16S rDNA sequencing of bacteria with active CM*

Selected bacteria that exhibited activity were subjected to 16S rDNA sequencing. Bacteria were grown in nutrient broth at 37^o^C with constant shaking. Bacteria were then pelleted by centrifugation and subjected to DNA extraction using QIAGEN DNA extraction kit, as detailed in the manufacturer’s instructions (Akbar et al., 2019). The extracted bacterial DNA was amplified using 16S amplification using Taq DNA Polymerase 2X-preMix and a pair of 16S rDNA Universal primers; 27F (5’-AGAGTTTGATCCTGGCTCAG-3’) forward primer and 1492R (5’-GGTTACCTTGTTACGACTT-3’) reverse primer, with conditions: 1 cycle at 95oC for 5 minutes, amplification step; 30 cycles (i) 95^o^C for 30 seconds, (ii) 55^o^C for 30 seconds, and (iii) 72^o^C for 1 minute and final step; 1 cycle at 72^o^C for 5 minutes. Gel electrophoresis was conducted using 1% agarose gel in 1x Tris-acetate-EDTA (TAE) buffer at 100 V for 40min. Amplified DNA was sequenced via Sanger sequencing. The nucleotide sequences obtained were aligned using ChromasPro software and were then blasted into the National Center for Biotechnology Information (NCBI) database for Standard Nucleotide BLAST, for 16S RNA sequences, to obtain the identity of the bacteria with maximum percentage nucleotide match (Akbar et al., 2019). 

*IC*_50_ *determination of active CM through MTT cell viability assay*

The IC_50_ value of active CM was determined using MTT (3-(4,5-dimethylthiazol-2-yl)-2,5-diphenyltetrazolium bromide) assay as previously described by (Akbar et al., 2019). Briefly, cells were grown to 70% confluency in 96-wells plates and treated with active CM at various concentrations; 2.5, 5.0, 10 and 20 µg/ml in growth medium. Post incubation, positive control cells were treated with 0.2% Triton X-100 for 30 min at 37°C, to induce 100% cell death. The percentage cell viability was calculated as follows:

% Cell viability = [(Absorbance sample – Absorbance blank)/ (Absorbance negative control – Absorbance blank)] X 100

*Identification of molecules(s) exhibiting anticancer activity through Liquid chromatography-mass spectrometry (LC-MS)*

Active CM were subjected to LC-MS analysis as described previously (Ali et al., 2017). Molecules were extracted from active CM through solvent extraction using chloroform in 1:3 ratio of chloroform to CM. The molecules dissolved in chloroform were subjected to evaporation and resuspended in 1:1 ratio of methanol to type 1 water. The samples were subjected to Agilent 1290 Infinity LC system coupled to Agilent 6520 Accurate-Mass Q-TOF mass spectrometer with dual ESI. The molecules were separated through Agilent Zorbax Eclipse XDB-C18 column with a particle size of 3.5 micron and Narrow-Bore of 2.1x150mmat 25oC using solvent A (0.1% formic acid in water) and solvent B (0.1% formic acid in Acetonitrile) for a total run time of 30 minutes. The separated molecules were then ionized by means of ESI + jet stream ion mode with the QQQ analyzer, with parameters: capillary voltage at 4500 V, sheath gas flow at 8 L per min, fragmentor voltage 135 V, gas temperature at 350ºC, gas flow at 8 L per min, and nebulizer gas at 40 psi and detector used was MCP Microchannel Plate detector, while the blank expended after each sample was of composition 50% MeOH + 50% MiliQ water. Chromatogram were generated from LC-MS which were used to determine the identity of the molecules from the Metlin_AM_PCDL-N-170502.cdb database. SciFinder database was then used to determine whether the identified molecules had previously reported biological activities.

## Results

*Various bacteria were isolated from faeces and gastrointestinal tract of C. livia*

Several Gram-positive and Gram-negative bacteria were isolated from the faeces and gastrointestinal tract of C. livia (Table 1). Gram-positive bacteria isolated were coagulase negative Staphylococcus spp., Gram positive bacilli, and Lysinibacillus boronitolerans, while the Gram-negative bacteria were Escherichia coli, and Pseudomonas aeruginosa. While from the gastrointestinal tract, Gram-positive bacteria; Gram positive bacilli, Lysinibacillus fusiformis, Lysinibacillus boronitolerans, coagulase negative Staphylococcus spp., Lysinibacillus sphaericus, Streptococcus group B, and Streptococcus saprophyticus and Gram-negative bacteria were Escherichia coli, and Sphingobacterium multivorum. As CLF05 and CLG01 showed potent activity, 16S rDNA sequencing was performed on these two bacteria. The result revealed that CLF05 and CLG01 are Bacillus cereus ATCC 14579 and Bacillus velezensis strain FZB42, respectively (Table 1).

*CM from faecal sample of C. livia inhibited HeLa cell growth*

**Figure 1:**
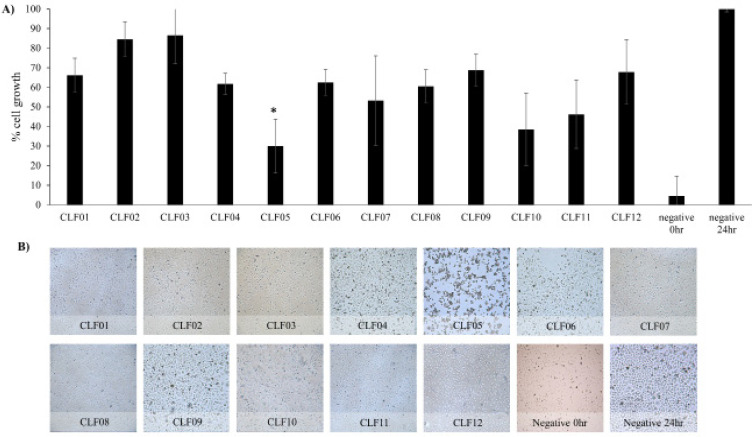
Growth Inhibition Effect of Conditioned Media (CM) Prepared from Bacteria Isolated from the Faecal Sample of Pigeon against HeLa Cells. A) Semi-confluent HeLa cells were incubated with CM and growth inhibition was determined as described in Materials and Methods. B) Using an inverted microscope, images of the cells were taken at x250. The results are representative of several experiments performed in duplicate. P value was determined using two sample T‐test, two‐tailed distribution, (*) is <0·05. Negative control (0h) is number of cells at the beginning of experiment, while negative control (24h) is untreated cells. The data are compared between negative control (24h) and CLF/CLG. Detailed CLF and CLG nomenclature is described in Table 1

**Figure 2:**
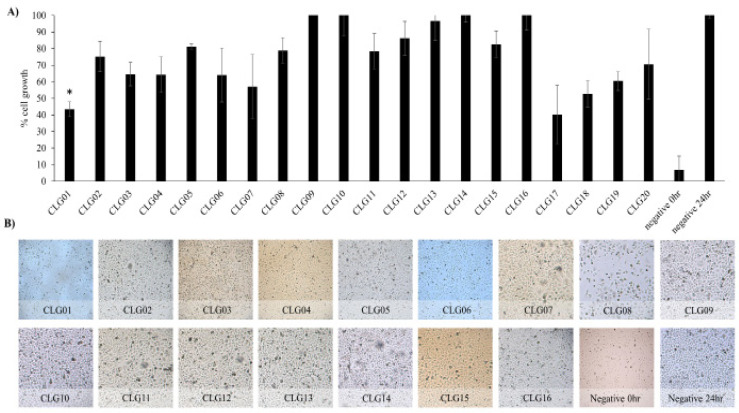
Growth Inhibition Effect of CM Prepared from Bacteria Isolated from the Gastrointestinal Ttract of Pigeon against HeLa cells. A) Semi-confluent HeLa cells were incubated with CM and growth inhibition was determined as described in Materials and Methods. B) Using an inverted light microscope, images of the cells were taken at x250. The results are representative of several experiments performed in duplicate. P value was determined using two sample T‐test, two‐tailed distribution, (*) is <0·05. Negative control (0h) is number of cells at the beginning of experiment, while negative control (24h) is untreated cells. The data are compared between negative control (24h) and CLF/CLG. Detailed CLF and CLG nomenclature is described in Table 1

CLF05, CLF07, CLF10 and CLF12 prepared from Bacillus cereus, E. coli, P. aeruginosa and E. coli, inhibited growth of HeLa by 70.0, 46.8, 61.5 and 53.7% respectively (Figure 1A). While remaining CM affected the cellular growth of HeLa by less than 40%. CLF01, CLF02, CLF03, CLF04, CLF06, CLF08, CLF09 and CLF11 showed 66.1, 84.5, 86.5, 61.8, 62.5, 60.5 and 67.8% HeLa cell growth respectively. The microscopic images supported the graphical representation of results (Fig. 2B). Moreover, CLF05 affected the morphology of HeLa cells as compared to the negative control cells.

*CM from gastrointestinal of C. livia inhibited the growth of HeLa cells*

CLG01, CLG07, CLG17 and CLG18 prepared from Bacillus velezensis, L. sphaericus, Streptococcus group B and S. multivorum inhibited growth of HeLa cells by 56.5, 43.0, 59.8 and 47.3% respectively (Figure 2A). However, CLG02, CLG03, CL04, CLG05, CLG06, CLG08, CLG09, CLG10, CLG11, CLG12, CLG13, CLG14, CLG15, CLG16, CLG19 and CLG20 showed 75.2, 64.6, 6.3, 81.2, 64.1, 78.9, 100, 100, 78.5, 86.3, 96.7, 100, 82.6, 100, 60.4 and 70.6% HeLa cell growth respectively. Moreover, the microscopic images depicted similar results as the graphical representation (Figure 2B).

*CLG01 and CLF05 produced damage to HeLa cells*

Microscopic images for HeLa cells treated with CLF05 showed change in morphology and the cells appeared round, as compared to untreated cells (negative control), albeit no LDH release was determined. In contrast, CM for faeces depicted morphology similar to the negative control. Additionally, wells containing cells treated with CLF05 remained stained similar to the positive control. 

*Concentrated CM inhibited growth of cancer and normal cells*

**Figure 3:**
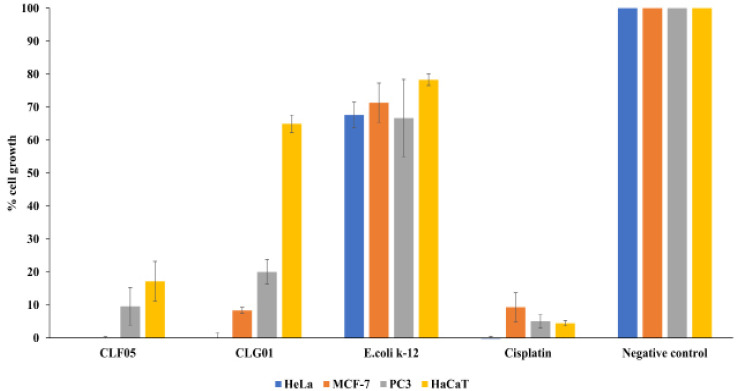
CLF05 and CLG01 Exhibited Effects against Cells at 10µg/ml. A) Semi-confluent HeLa, MCF-7, PC3 and HaCaT cells were incubated CLF05 and CLG01 as described in Materials and Methods. Untreated cells were considered as 100% and effects of CLF05 and CLG01 are presented as relative change. The results are representative of several experiments performed in duplicate. Note that CLG01 exhibited potent effects against all cancer cell lines tested, except normal Hacat cells; while CLF05 showed effects against all cells tested. Control CM from E. coli K-12 showed no effects against any of the cell lines tested

**Figure 4:**
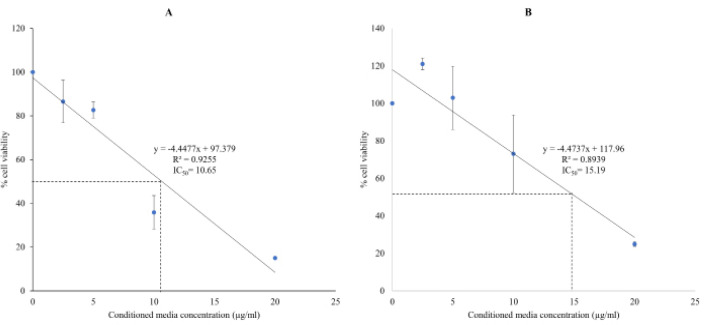
IC_50_ Concentration of CLF05 and CLG01 against HeLa Cells Using MTT Assay. Cells were incubated with various concentrations of CLF05 and CLG01 and cell survival determined using MTT assays as described in Materials and Methods. Note that both CLF05 and CLG01 exhibited IC50 below 20 µg/ml. The results are representative of several experiments performed in duplicate

At a concentration of 10µg/ml, CLF05 inhibited growth of HeLa, MCF-7, PC3 and HaCaT cells by 100, 100, 90.4 and 82.8% respectively, while CLG01 inhibited growth of aforementioned cells by 100, 91.6, 80.0 and 35.1% respectively (Figure 3). The quantitative data were further supported by microscopic images obtained post-treatment of normal and cancer cells. MTT assay results revealed that CLF05 and CLG01 affected viability of HeLa cells at an IC_50_ concentration of 10.65 and 15.19 µg/ml respectively (Figure 4).

*CLF05 and CLG01 possess 111 and 71 molecules*

CLF05 and CLG01 were subjected to LC-MS. Figure 6 shows spectra (negative and positive ion polarity) of molecules detected from CLF05 and CLG01. A total of 111 molecules were detected from CLF05, out of which 54 molecules were identified (Table 2 and Supplementary Table 1), while the remaining molecules are unidentified and potentially novel. Moreover, out of the 54 identified molecules, only 18 had reported biological activity (Supplementary Table 1) while one molecule had reported anticancer effects; namely dihydroxymelphalan. The remaining 57 unidentified molecules from CLF05, had limited information including retention time, molecular mass, and molecular formula (Supplementary Table 2). For CLG01, a total of 71 molecules were detected from HSG16, out of which 6 molecules were identified (Table 2 and Supplementary Table 1), while the remaining molecules are unidentified. Out of 6 identified molecules, one molecule had reported biological activity (Supplementary Table 1) and none had reported anticancer activity. The remaining 65 unidentified molecules from CLG01 depicted limited information including retention time, molecular mass, and molecular formula (Supplementary Table 2).

**Table 1:** The Bacteria Species Isolated from the Faeces and Gastrointestinal Tract of pigeon

	Gram stain	*Bacteria*
Pigeon faecal		
CLF01	Gram-negative	*Escherichia coli*
CLF02	Gram-positive	Coagulase Negative* Staphylococcus *spp
CLF03	Gram-negative	*Escherichia coli*
CLF04	Gram-positive	Gram positive *bacilli*
CLF05	Gram-positive	Gram positive* bacilli*
CLF06	Gram-positive	*Lysinibacillus boronitolerans*
CLF07	Gram-negative	*Escherichia coli*
CLF08	Gram-negative	*Escherichia coli*
CLF09	Gram-negative	*Escherichia coli*
CLF10	Gram-negative	*Pseudomonas aeruginosa*
CLF11	Gram-negative	*Escherichia coli*
CLF12	Gram-negative	*Escherichia coli*
Pigeon gut		
CLG01	Gram-positive	Gram positive* bacilli*
CLG02	Gram-positive	*Lysinibacillus fusiformis*
CLG03	Gram-positive	Gram positive* bacilli*
CLG04	Gram-positive	*Lysinibacillus boronitolerans*
CLG05	Gram-negative	*Escherichia coli*
CLG06	Gram-positive	Coagulase Negative* Staphylococcus *spp
CLG07	Gram-positive	*Lysinibacillus sphaericus*
CLG08	Gram-positive	*Lysinibacillus sphaericus*
CLG09	Gram-positive	Gram positive* bacilli*
CLG10	Gram-positive	*Lysinibacillus boronitolerans*
CLG11	Gram-positive	*Lysinibacillus fusiformis*
CLG12	Gram-positive	*Lysinibacillus fusiformis*
CLG13	Gram-negative	*Escherichia coli*
CLG14	Gram-negative	*Escherichia coli*
CLG15	Gram-negative	*Escherichia coli*
CLG16	Gram-positive	*Lysinibacillus sphaericus*
CLG17	Gram-positive	*Streptococcus group B*
CLG18	Gram-negative	*Sphingobacterium multivorum*
CLG19	Gram-positive	*Streptococcus saprophyticus*
CLG20	Gram-positive	Coagulase negative* staphylococcus aureus*

**Table 2:** The Molecules Detected, Identified and Unidentified from the Conditioned Media Prepared from Bacteria Isolated from the Faecal Matter and Gastrointestinal Tract of *C. livia* through LC-MS. The conditioned media prepared were subjected to chloroform extraction and the extracts were subject to LC-MS analysis. The spectre generated were searched in the METLIN library in order to reveal the potential identity of the detected molecules. To assess whether those identified molecules had previously reported biological activity, they were searched in the SciFinder database

Bacteria	Number of molecules				
	Detected	Identified	Reported activity	Anticancer activity	Unidentified
*Bacillus cereus* (CLF05)	111	54	18	1 Dihydroxymelphalan	57
*Bacillus velezensis *(CLG01)	71	6	1	-	65

**Figure 5:**
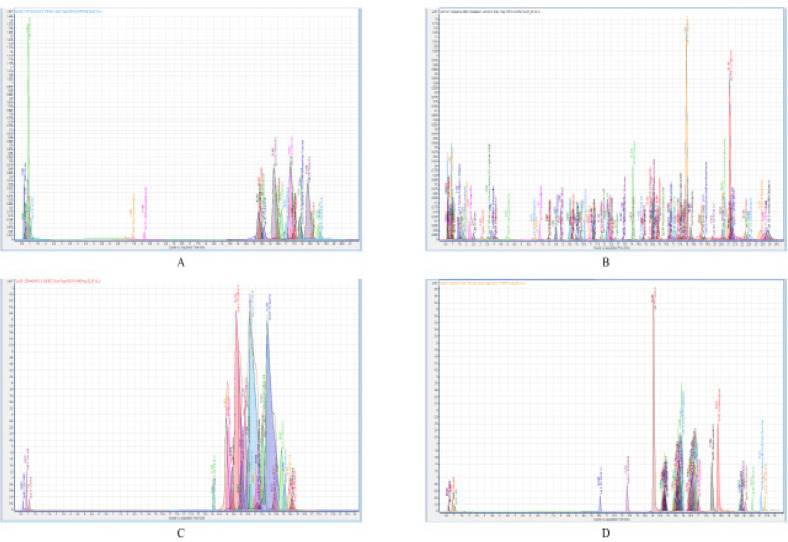
LC-MS Spectra of the Active CM, CLF05 (Bacillus Cereus) and CLG01 (Bacillus Velezensis). Briefly the CM was subjected to chloroform extraction and the extract was dried under pressure and dissolved in HPLC grade methanol for LC-MS analysis. [A: The spectrum of molecules detected for negative ion polarity from CLF05, B: the spectrum of molecules detected for positive ion polarity from CLF05, C: The spectrum of molecules detected for negative ion polarity from CLG01 and F: the spectrum of molecules detected for positive ion polarity from CLG01].

## Discussion

There is continued rise in morbidity and mortality associated with cancer, despite the wide range of available treatment options, hence it is imperative to discover and develop new anticancer agents. Our previous work highlighted that animals that thrive in polluted environments, such as crocodiles, cockroaches, water monitor lizards and snakes, may possess anticancer and antibacterial mechanisms to ward off disease (Khan et al., 2019; Jeyamogan et al., 2017; Mosaheb et al., 2018; Soopramanien et al., 2019). In addition to the animal lysates, the faecal and gut microbiota is of particular interest to us, as the gut microbiota is known to play an important role in regulating the behaviour and health of its host (Jeyamogan et al., 2017; Heyde and Ruder, 2015; Alarcón et al., 2016). Anticancer effects from avian species remain unexplored and were the subject of present study. A repertoire of bacteria was isolated from both the faecal and gut microbiota of C. livia. 

The results revealed that B. cereus and B. velezensis exhibited potent effects against cancer cell lines tested. CM from CLF05 (B. cereus) and CLG01 (B. velezensis) inhibited growth of HeLa cells by more than 40%. Moreover, they inhibited almost 100% cell growth of cancer cells at a concentration of 10µg/ml and affected the viability of HeLa cells at IC50 concentrations of 10.65 and 15.19 µg/ml, respectively. Furthermore, microscopic images revealed that CLF05 and CLG01 produced damage to HeLa cells. Although LDH release was negligible but CM affected the integrity human cells indicating that cell death might have resulted from apoptosis. Thus, further experiments are needed to determine apoptosis in treated cells. 

Previous work has reported that Bacillus species produce metabolites with anticancer properties, which support our findings (Ferdous et al., 2018). Bioactives such as ε-Poly-L-lysine, Surfactin, Leodoglucomide B, Bacillistatins-1 and 2 and Mixirins A, B and C synthesised by bacteria have shown to inhibit growth and induce morphological changes in cancer cells (Arnold et al., 1979; Harris and Pierpoint, 2012; Pettit et al., 2009; Tareq et al., 2012; Wu et al., 2017). Previously, anticancer potential of metabolites secreted by B. cereus isolated from soil sample were tested against human liver cell lines and cancer cell lines (Kumar et al., 2014). The results revealed that metabolites from B. cereus was cytotoxic to cells with IC50 of 225.4 µg/mL, 152.2 µg/mL, and 152.2 µg/mL against HepG2, Hep2 and Chang liver cells, respectively. These findings suggest that metabolites released by B. cereus exhibit anticancer activity by inducing apoptosis in cancer cells (Kumar et al., 2014). 

The LC-MS revealed 111 molecules including dihydroxymelphalan. Dihydroxymelphalan, also known as melphalan is a widely used anticancer drug. It works by causing inter-strand cross-links in DNA strands; resulting in inhibition of DNA and RNA synthesis (Kuczma et al., 2016; Rahaman et al., 2018). Moreover, melphalan upregulates expression of reactive oxygen species which in turn triggers apoptosis in cancer cells by activating caspase-9 (Kuczma et al., 2016; Rahaman et al., 2018). Since melphalan detected in CM from CLF05 induces apoptosis, it supports our speculation that CLF05 induces damage in cancer cells through apoptosis. The other molecules that were elucidated are tazobactam-m1, citric acid, 3-furoic acid, 4-hydroxyphenylglyoxylate, diethanolamine, methionyl-hydroxyproline, bifenazate, diethyltoluamide, metabutethamine, rivastigmine, tropicamide, xylylcarb, and daimuron. These molecules exhibited activities ranging from antimicrobial, hypolipidemic, cholinesterase inhibitor, to inhibitor of fatty acid oxidation, however, their anticancer effects need to be determined (Halstenson et al., 1994; El Baaboua et al., 2018; In et al., 2013; Hall et al., 1985; Stephens et al., 1985; Leung et al., 2005; Deblander et al., 2015; Asano et al., 1997; Alberts et al., 1979; Allen et al., 1970; Ochiai et al., 2007; Sudakin and Osimitz, 2010; deShazo and Nelson, 1979; Khoury et al., 2018; Manny et al., 2001). Similarly, none of the molecules from CLG01 were reported to possess anticancer effects and this will be determined in future studies. Of note, 57 molecules detected from CLF05 and 65 molecules detected from CLG01 respectively remain unknown and are potentially innovative anticancer agents. Moreover, only aerobic culturable bacteria were isolated in this study and future studies are needed to isolate anaerobic bacteria and unculturable bacteria, that might also be a principal resource of anticancer molecules. 

In conclusion, for the first time, we have showed that gut and faecal microbiota of avian species are a potential source of anticancer properties. We have elucidated various molecules that could serve as possible drug leads; but further research is needed to achieve these prospects. It will be imperative to investigate anticancer effects of bacteria against other cancer cells. Our results further augment our speculation that animals residing in unsanitary habitats, that are detrimental to humans, maybe in fact a large unexploited repertoire for innovative pharmaceutical drugs.

## Author Contribution Statement

RS and NAK conceived the idea. MS and BNHMN sourced the animals and carried out dissections under the supervision of KS. MS and BNHMN carried out all experiments under the supervision of RS and NAK. MS carried out LC/MS analyses under the supervision of RS. MS prepared the first draft of the manuscript. NAK and RS corrected the manuscript. All authors approved the manuscript.

## Declarations

Ethics Approval and Consent to Participate: This article does not contain any studies with human participants. The use of animals was approved by Sunway Research Ethics Committee (Research Ethics Approval Code: PGSUNREC 2019/023). We also confirm that all experiments were performed in accordance with relevant guidelines and regulations.
